# Correlating Molecule Counts with Stress Granule Size Using Novel Platinized Carbon Nanopore Electrodes

**DOI:** 10.1021/acs.analchem.6c01191

**Published:** 2026-06-02

**Authors:** Yue Wang, Chaoyi Gu, Hui Gu, Yingjie Zhao, Dengchao Wang, Andrew G. Ewing

**Affiliations:** † Department of Chemistry and Molecular Biology, 3570 University of Gothenburg, Gothenburg 41390, Sweden; ‡ School of Chemical Sciences, University of Chinese Academy of Sciences, Beijing 10049, China; § Department of Chemistry and Chemical Engineering, 12518 Hunan University of Science and Technology, Xiangtan 411201, China

## Abstract

Stress granules (SGs) are membraneless dynamic structures formed through liquid–liquid phase separation (LLPS) under stress conditions; they can pause nonessential translation and reprogram gene expression to cope with stress, thus being crucial for cell survival. This study pioneers an oxidative stress model that links SG size to redox regulation. Novel platinized carbon nanotube nanopipettes (Pt-CNTNPs) were employed for SG impact electrochemical cytometry (SGIEC) and intracellular SGIEC (ISGIEC) measurements, correlating H_2_O_2_ content with SG dimensions through a size-exclusion mechanism. Gaussian fitting quantified the distribution of H_2_O_2_ molecule number, and data from PEG8000 solutions demonstrated that intracellular-extracellular content number disparities are likely attributable to macromolecular crowding. All of the results confirm that H_2_O_2_ generation in SGs is governed by an interfacial electric field. By establishing a quantitative SG-mediated redox framework, this study provides novel insights into LLPS and opens avenues for targeted therapies and biomimetic materials.

## Graphical abstract

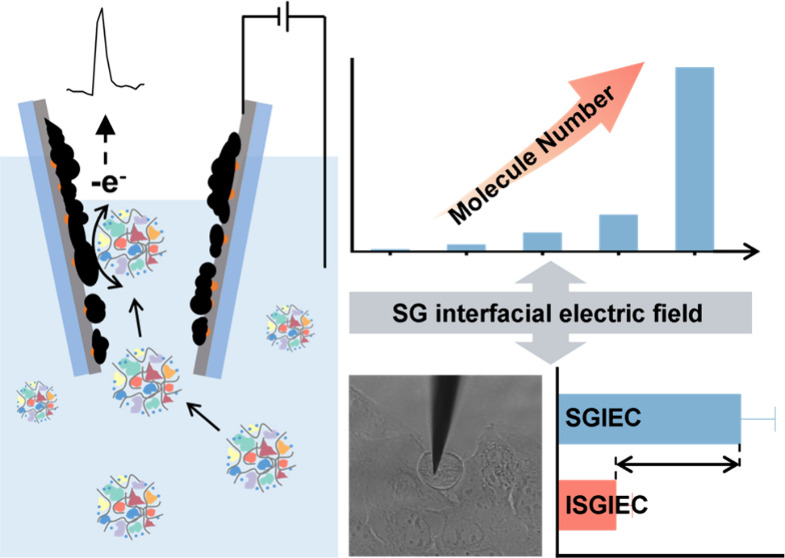

## 1 Introduction

Stress granules (SGs) are dynamic nonmembrane organelles (ranging from 100 to 2000 nm) combined by mRNA-binding proteins, untranslating messenger ribonucleoproteins, and translation initiation factors through liquid–liquid phase separation (LLPS) when facing external stresses such as heat/cold shock, oxidative stress, UV irradiation, or viral infection.^1–3^ They play an important role in affecting mRNA translation and degradation, selective sequestration of viral RNA, and surveillance of aberrant RNA to enhance cellular fitness in adverse environments.^4–6^ Research on SGs has revealed fundamental principles governing cellular stress adaptation, which not only decodes the molecular logic of stress-induced translation arrest and mRNA sorting but also directly links SG malfunction with the pathogenesis of neurodegenerative diseases.^7^ For instance, targeting the SG disassembly mechanism (such as modulating G3BP1 phosphorylation) emerges as a hopeful therapeutic strategy to reduce proteotoxicity in Alzheimer’s and Parkinson’s diseases.^8,9^

Reactive oxygen species (ROS) such as hydrogen peroxide (H_2_O_2_) have been shown to be formed at SGs, and these ROS appear to serve significant roles as both signaling molecules in stress responses and modulators of other organelle dynamics like lysosomes and mitochondria.^10–12^ ROS have been discovered in SGs,^10^ where catalase was used to suggest that H_2_O_2_ was the main ROS present, and subsequently Gu et al. used platinized small disc electrodes to carefully characterize the response, showing definitive evidence for H_2_O_2_.^13^ Quantifying H_2_O_2_ concentration in a single SG is important to unravel the formation mechanism of H_2_O_2_ inside SGs; thus, it is necessary to develop a nanoscale tool to determine the SG size and its content to accurately establish their relationship.

Traditional methods for SG analysis include fluorescent labeling, live-cell imaging, proteomics, and CRISPR screening,^14–16^ which rely on labeled biomarkers or population-level analysis, mask cellular heterogeneity, and fail to capture the real-time chemical microenvironment during SG assembly and disassembly. Our group has utilized fluorescence confocal microscopy in U2OS cells stably expressing the canonical SG marker, G3BP1-GFP, to confirm the presence of these structures and employed the fluorogenic probes to detect the production and presence of hydrogen peroxide within the separated SGs.^13^ Compared to those approaches, single-cell electrochemical techniques enable label-free, real-time monitoring of redox states, ion fluxes, and molecular interactions at the single-cell/organelle level using nanoscale electrodes with high temporal-spatial resolution. For example, vesicle impact electrochemical cytometry (VIEC) with carbon nanopipettes has been used to characterize transmitters in single vesicles, providing correlations of vesicle content and measured kinetics with its size.^17^

In this study, we developed a novel carbon nanotube-decorated nanopipet sensor with internal modification of platinum black. By precisely controlling the orifice diameter of the electrode, we established a quantitative correlation between the SGs’ geometric dimensions and their electrochemical characteristics using SG impact electrochemical cytometry (SGIEC). The relationship between the H_2_O_2_ molecular number and the size of the SGs is consistent with a mechanism of H_2_O_2_ generation at the surface of the SG. Meanwhile, we observe a difference between SG content from SGIEC in bulk solution and intracellular SGIEC (ISGIEC) detection, which further emphasizes the key function of an electric field at the SG-solution interface in H_2_O_2_ production within SGs in a living cell.^13^

## 2 Materials and Methods

### 2.1 Chemicals

Polyethylene glycol 8000 was bought from Fisher Scientific. All the other chemicals were of analytical grade and purchased from Sigma-Aldrich. The lysis buffer consisted of 50 mM Tris-HCl, 100 mM potassium acetate, 2 mM magnesium acetate, 0.5 mM DTT, 50 μg/mL of heparin, 0.5% NP-40, and 1 EDTA-free protease inhibitor mini tablet. The isotonic saline buffer consisted of 150 mM NaCl, 5 mM KCl, 1.2 mM MgCl_2_, 2 mM CaCl_2_, and 5 mM glucose in 10 mM HEPES at pH 7.4. All the solutions were prepared with 18 MΩ·cm water from Purelab Classic purification (ELGA, Sweden).

### 2.2 Fabrication of the Electrode

#### 2.2.1 Preparation of Different-Sized Carbon Nanopipettes (CNPs) and Au Nanoparticles Catalyzed Carbon Nanotube Nanopipettes (Au NPs-CNTNPs)

A laser puller (P-2000, Sutter Instruments) was used to make the bare quartz nanopipettes. Different tip size (50, 200, 500, 750, 1000, and 1500 nm) nanopipettes were pulled from quartz capillaries (Q100–50–7.5, o.d. = 1 mm, i.d. = 0.5 mm and length = 7.5 cm) by suitable parameters (HEAT = 680, FIL = 3, VEL = 22, DEL = 135, PUL = 130/125/120/115/110/105). The CNPs were made through the chemical vapor deposition (CVD) method to deposit a carbon layer inside the bare nanopipettes; methane was used as the carbon source, and argon was used as the protective gas (ratio is 5:8). The temperature was 980 °C, and the deposition time was 25 min. For the preparation of Au nanoparticles catalyzed carbon nanotube nanopipettes (Au NPs-CNTNPs), the quartz nanopipettes were initially immersed in 0.1 μM chloroauric acid of ethanol solution for 15 min. After being dried in the air for 6 h, the treated nanopipettes were subsequently coated with a layer of carbon nanotubes via the CVD method. The gas ratio was 1/1. the temperature was 970 °C, and the deposition time was 15 min.

#### 2.2.2 Preparation of Both Platinized CNP and Au NPs-CNTNPs

The platinization procedure was according to the previous report.^8^ The platinizing solution contained 1.5% H_2_PtCl_6_ and 80 μM lead­(II) acetate in PBS (pH 7.4). Four voltammetric cycles were applied from 0 to −400 mV at 400 mV/s to an electrode to activate the inner surface. Then an −80 mV (vs Ag/AgCl reference) electrode bias was used in the same solution with a CHI electrochemical analyzer (CH Instruments, Inc., Austin, TX, USA). The deposition was stopped before a sharp change in the slope of the amperometric curve occurred. A slightly different time was used depending on the size of the electrode. Two kinds of platinized nanoelectrodes were tested in H_2_O_2_ solution of different concentrations (from 0 μM to 5 mM) in PBS (pH 7.4) by cyclic voltammetry (0 to 0.8 V vs Ag/AgCl, 0.1 V/s) using a CHI electrochemical analyzer.

To ensure the highest degree of accuracy for the determination of tip diameters, we used high-resolution TEM imaging as our primary validation tool. We performed TEM on many electrodes fabricated under the same parameters to confirm that our fabrication process is highly reproducible and that the inner diameters remain stable.^18,19^

### 2.3 U2OS Cell Culture

Human bone osteosarcoma epithelial (U2OS) cells were cultured in high-glucose Dulbecco’s Modified Eagle Medium (DMEM) containing 10% fetal bovine serum (FBS), 1% penicillin-streptomycin, and 1 μg/mL of puromycin. The cells were maintained in TC-treated T75 flasks (from Sarstedt, Sweden) under a humidified atmosphere of 5% CO_2_ at 37 °C until a confluent monolayer was achieved.

### 2.4 Isolation of SGs from U2OS Cells

The isolation procedure was carried out according to a previous report to obtain a granule-enriched fraction including primarily SGs and other proteins.^10,13^ Consistent with literature benchmarks for SG isolation, this preparation achieved a 5-fold to 10-fold enrichment of SGs, as validated by the fold-increase of the SG marker protein G3BP1 in the final fraction relative to the whole-cell lysate.^20^ To stress the cells, U2OS cells were initially exposed to 100 μM arsenite for 1 h and centrifuged at 300 *g* for 10 min. The resulting pellet was reconstituted in 1 mL of SG lysis buffer and flash-frozen. Cellular lysis was performed by passing the suspension 7 times through a 25 G 5/8 needle on ice, followed by centrifugation at 1,000 × g for 10 min. The supernatant enriched with SGs was subjected to ultracentrifugation at 18,000 *g* for 20 min to isolate the SG pellet. This pellet was then resuspended in 1 mL of fresh lysis buffer and centrifuged again under the same ultracentrifugation conditions. A temperature of 4 °C should be kept in all centrifugation steps.

### 2.5 SG Impact Electrochemical Cytometry (SGIEC) Measurement

The SG pellets were resuspended in 100 μL of lysis buffer or 5% PEG8000 modified lysis buffer for electrochemical detection. The working potential was applied by an Axopatch 200B potentiostat (Molecular Devices, Sunnyvale, CA) at +700 mV vs a handmade Ag/AgCl reference electrode. The output was converted into digital form by Digidata1440A (Molecular Devices) at a frequency of 10 kHz and filtered at 2 kHz with a 4-pole Bessel filter. All amperometric measurements were carried out by employing a two-electrode system within a firmly grounded Faraday cage on the vibration isolation lab table (TMC, Eabody, MA, USA).

### 2.6 Intracellular SGIEC (ISGIEC) Measurement

The U2OS cells were equilibrated in isotonic saline buffer in electrochemical experiments. An inverted microscope (Olympus IX81) equipped with 10× and 40× objectives was used to observe the U2OS cells. The Au NPs-CNTNP, mounted on a precision patch-clamp micromanipulator (Burleigh Instruments PCS-5000, USA), was first positioned near a U2OS cell and subsequently penetrated the membrane of the targeted cell, with real-time current recording during the process. The output form was the same as the SGIEC measurement.

### 2.7 Data Analysis

Both the SGIEC and ISGIEC data were exported as .txt format files via MATLAB (The MathWorks, Inc.) and subsequently analyzed using Igor Pro software (version 6.22, Wavemetrics, Lake Oswego, OR). Following automated peak identification, all traces were inspected to eliminate false events. Quantified parameters—including the number of molecules released per stress granule and the half-life (*t*_1/2_)—were summarized with median values calculated for each experimental group. For intergroup comparisons, the averaged medians of these parameters were statistically evaluated using either one-way ANOVA (≥3 groups) or the Mann–Whitney rank sum test (unpaired, two-tailed; for two groups) in GraphPad Prism 10 (GraphPad, La Jolla, CA). Significance thresholds were defined as ****p < 0.0001, ***p < 0.001, **p < 0.01, *p < 0.05, and ns (not significant) for *p* > 0.05.

## 3 Results and Discussion

Li et al. reported a platinization method with CNPs for the detection of ROS/RNS inside human breast cells.^21^ On the basis of this method, we deposited the Pt-black within our Au nanoparticles-catalyzed carbon nanotube nanopipettes (CNT-NPs)^22^ to obtain cavity Pt nanoelectrodes as shown in Figure 1A. Because of the larger specific surface area and higher surface activity of carbon nanotubes (CNTs), Pt black prefers to grow at CNTs along the inner wall rather than becoming aggregated platinum that blocks the pipet tip, thereby forming a cavity suitable for SGs to enter and react. This is also consistent with the TEM characterization in Figure 1B. The platinized carbon nanotube nanopipettes (Pt-CNTNPs) remain open at the tip and show oxidization peaks for a series of H_2_O_2_ concentrations (Figures 1C,D, and S1). While electrochemical measurements provide functional verification of the electrode geometry, they rely on geometric assumptions that may introduce uncertainty. To validate our electrode dimensions, we performed high-resolution TEM imaging on many electrodes fabricated under identical parameters in Figure S1. The high reproducibility of our fabrication process ensures consistent inner diameters across all devices. By controlling the deposition time and deposition current, we have ensured that a cavity is still retained in each electrode, as illustrated in Figure 1C. Generally, the peak for H_2_O_2_ oxidation is at 0.3 V, and a small peak is observed for platinum oxidation at 0.7 V. As this latter peak is constant, it should not affect the transient measurement of the SGs. It should be noted that CNPs and CNT-NPs do not respond to H_2_O_2_, and platinized CNPs (Pt-CNPs) are typically fully filled disk electrodes without an internal cavity. A comparison of the calibration curves for H_2_O_2_ at the two types of electrodes is displayed in Figure 1E, showing better H_2_O_2_ sensing performance for our novel electrode. Moreover, the anodic currents maintained about 90% of the initial current within 4 h and dropped to 50% on the third day after fabrication at the Pt-CNTNPs, which is much more stable than Pt-CNPs toward H_2_O_2_ oxidation (Figure 1F).

**Figure 1:**
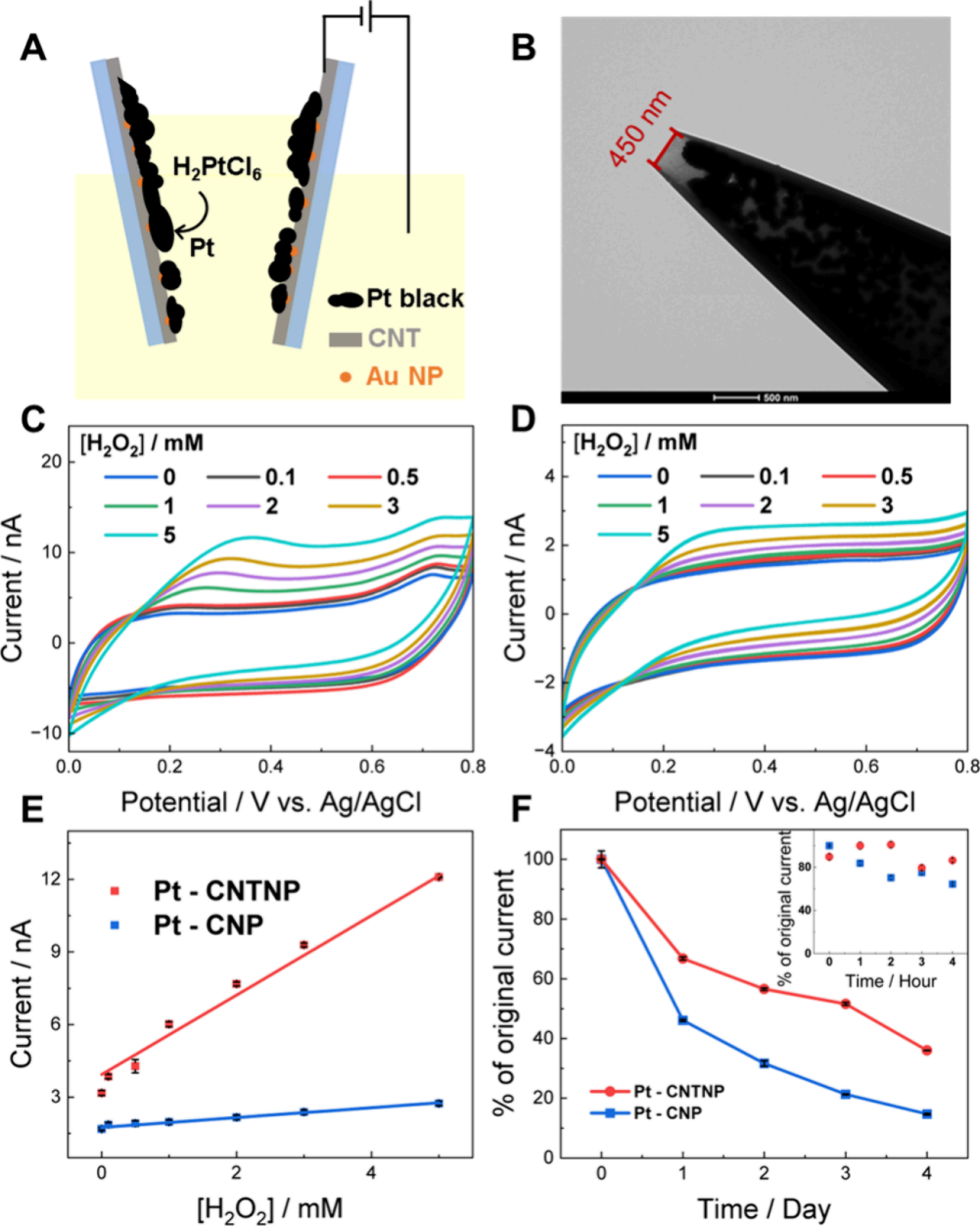
Fabrication and characterization of novel Pt black-Au NP-modified carbon nanotube nanopipettes (Pt-CNTNPs) compared to platinized carbon nanopipette (Pt-CNP) electrodes. (A) Scheme showing fabrication of Pt-CNTNPs. (B) TEM image of the Pt-CNTNPs with a diameter of 450 nm. Cyclic voltammograms with different concentrations of H_2_O_2_ in 10 mM PBS buffer at Pt-CNTNPs (C) and ordinary Pt-CNPs (D). (E) Calibration curves for H_2_O_2_ obtained from Pt-CNTNP (C) and Pt-CNP (D). (F) Stability of H_2_O_2_ oxidized currents at two kinds of Pt electrodes in PBS solution with 5 mM H_2_O_2_. The scan rate is 0.1 V/s. The data for the carbon nanotube characterization was gathered from the electrode shown in (B).

Figure 2A presents a diagram of SGIEC measurement with a hollow Pt-CNTNP in lysis buffer. This design has a size-exclusion mechanism so that only the SGs with a diameter smaller than the electrode aperture can diffuse in and react in the electrode cavity. Diameters of electrode apertures used for SGIEC were 50, 200, 500, 750, 1000, and 1500 nm. TEM characterization of these electrodes and statistical histograms of the size are shown in Figure S2. Representative amperometric profiles recorded at +700 mV (vs Ag/AgCl reference) for different diameters of Pt-CNTNPs are shown in Figure 2B. The solution contains the SGs from U2OS cells, and thus the oxidation peaks only come from H_2_O_2_ in SGs.^10,23^ For single collision analysis, the number of H_2_O_2_ molecules detected in each oxidized peak was calculated quantitatively by Faraday’s law: *Q* = *n* × *N*_molecules_ × *F*/*N*_*A*_, where *Q* is the integral of the peak current over time for a single peak, *n* is the number of electrons transferred in the oxidation reaction (for H_2_O_2_, *n* = 2), F is the Faraday constant (96485 C/mol), and *N*_*A*_ is the Avogadro constant (6.022 × 10^23^). As these measurements are based on SG impacts, some SGs might be adsorbed during these measurements; however, the inner surface of the electrode is orders of magnitude larger than the impacted area of an SG, and we only measure 10 s of collision events in each experiment, so this should be irrelevant. It is important to clarify that our carbon nanopipette electrodes function as integrative sensors. A nanopipette with a 200 nm inner diameter allows all SGs with diameters up to 200 nm to enter the sensing zone and collide with the platinized inner wall. Our measurements show that within this range, the detected SG population follows a characteristic Gaussian distribution.

**Figure 2:**
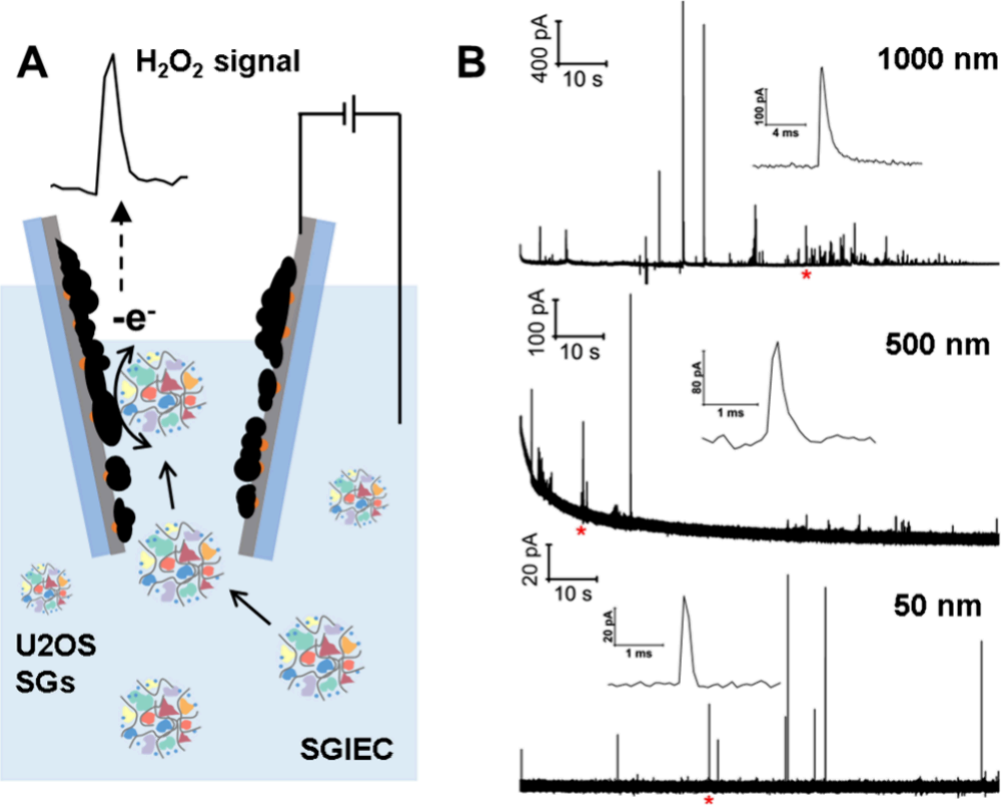
SGIEC measurement of SGs from U2OS cells using different sizes of Pt-CNTNPs. (A) Diagram of SGIEC measurement with hollow Pt-CNTNPs in lysis buffer. (B) Representative amperometric profiles recorded at +700 mV (vs Ag/AgCl reference) for Pt-CNTNPs (from top to bottom) with diameters of 1000, 500, and 50 nm Characteristic current transients with red asterisks are magnified in insets.

By summarizing the distribution frequency of the logarithmic values of *N*_molecules_, the 2D frequency distribution histograms are shown in Figure 3A; the related 3D frequency histograms and their Gaussian curves are shown in Figure 3B. For SGs smaller than 50 nm, 25% of them contain H_2_O_2_ numbers, *N*_molecules_, from 1 × 10^3.5^ to 1 × 10^4^. This ratio decreases as the SG size increases. As the larger nanopipette tip allows larger SGs to enter, the range of *N*_molecules_ increases from 1 × 10^6^ to 1 × 10^7^, as expected. The trend from small to large H_2_O_2_ amounts is more intuitive from the Gaussian distributions projected parallel to the *xz* plane (the Gaussian distribution fitting failed for the frequency histogram obtained using 1500 nm pipet tips). With increased diameter, the mean value of the Gaussian distribution increases accordingly, which again means that the larger SGs have a higher content of H_2_O_2_. Also, the value of σ (standard deviation) generally becomes larger (Figure S3) for larger nanopipette tips, indicating that the range of molecule number broadens due to the increase in the size range of SGs. The correlation between the average of the median values of *N*_molecules_ obtained from each SGIEC curve with the electrode tip size (Figure 3C) further confirms this trend. The *t*_1/2_ values for the peaks generally exhibit a positive correlation with the SG dimension; larger SGs contain a higher molecular count and need more time to be fully oxidized (Figure S4). This phenomenon fits a model where an interfacial electropolarization-dependent H_2_O_2_ generation mechanism occurs for SGs.^13^ The surface electric field governs the formation pathways of H_2_O_2_, leading to its predominant localization at the SG interface, so larger SGs exhibit increased electroactive surface area; thus the extended electrode contact duration during SG collisions amplifies charge transfer opportunities.

**Figure 3:**
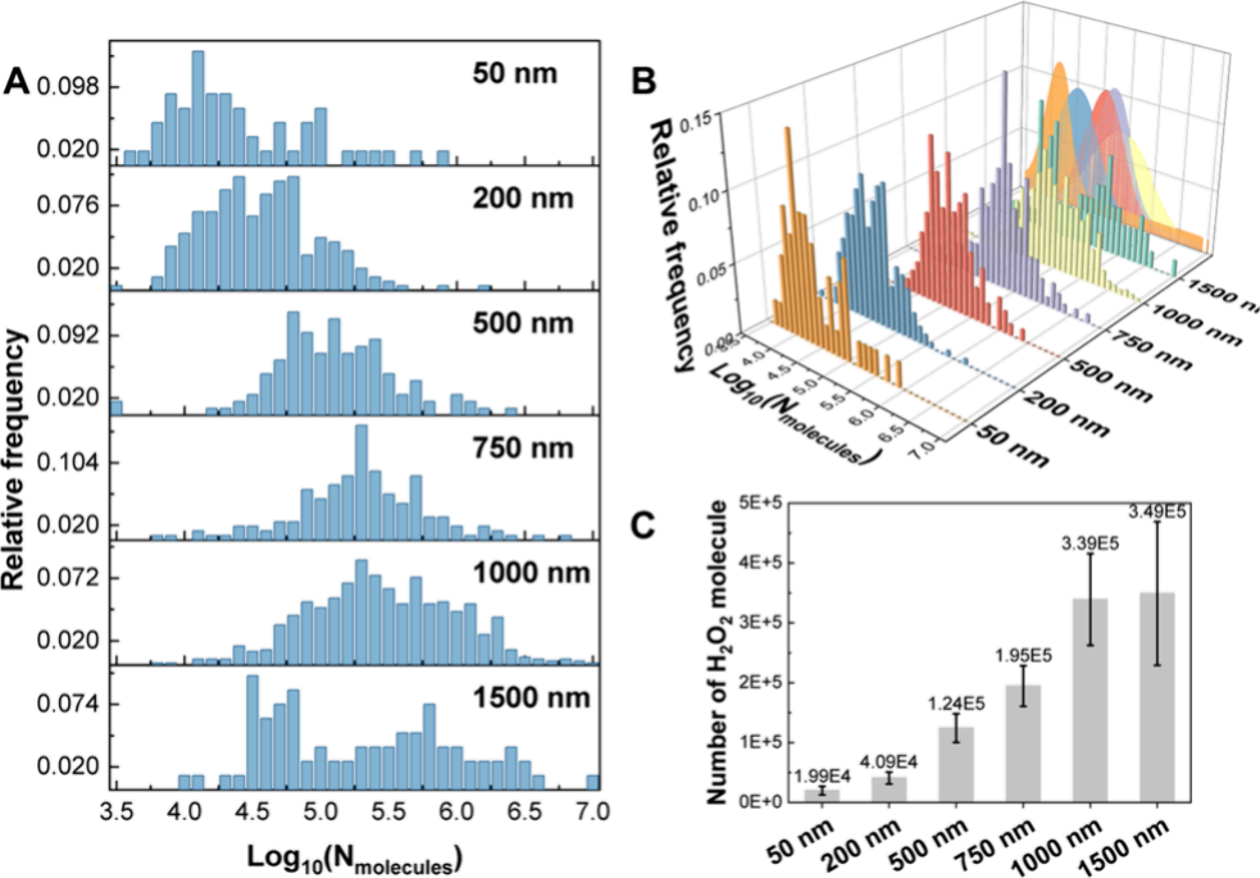
Histograms for the number of molecules obtained on six sizes of electrodes. (A) Related 2D frequency histograms for the number of molecules obtained over six sizes of electrodes (50, 200, 500, 750, 1000, and 1500 nm). (B) 3D frequency histogram for the number of molecules obtained on six sizes of electrodes (50, 200, 500, 750, 1000, and 1500 nm) with the relative Gaussian curves projected onto the *xz* plane. (C) Average *N*_molecules_ per electrode size. All these data were collected from six isolations of SGs from U2OS cells and 73 Pt-CNTNP electrodes.

It is worth noting that the number of molecules acquired by 1000 and 1500 nm electrodes is similar, and it is in accordance with the reported *N*_molecules_ (around 3 × 10^5^) using platinized carbon fiber nanotip electrodes.^13^ This implies that the 1000 nm electrode tip is capable of detecting all SGs, suggesting that the size of SGs obtained by stimulating with 100 uM arsenite for 1 h appears to be smaller than 1000 nm. A bimodal frequency histogram is obtained with the 1500 nm electrode (with a high relative frequency at 10^4.5^–10^5^ and a relatively low frequency at 10^5.0^–10^5.5^). This might indicate the simultaneous transport and collision of multiple different-sized SGs inside the cavity, so that multiple events could merge into one oxidation peak, which needs to be further investigated.

To determine the H_2_O_2_ content within SGs of specific size ranges, the experimental data were processed using a custom Python-based algorithm to perform nonlinear fitting via the Levenberg–Marquardt algorithm on the experimental data;^24^ the complete code is available in Supporting Information. Experimental data from large-scale electrodes were hypothesized to decompose into two components: the baseline profile corresponding to small-scale SGs and the characteristic Gaussian-shaped peaks representing *N*_molecules_ of SGs within these two size regimes. The formula for this composite model is $y_{\mathrm{mod}\mathrm{el}}(x)=\alpha \times f_{\mathrm{known}}(x)+A\times \exp\left(-\frac{(x-\mu {)}^{2}}{2\sigma^{2}}\right)$ , where α is the scaling factor of *f*_known_(*x*) and A, μ, and σ are amplitude, mean, and standard deviation of the Gaussian peak. Fitting results and the goodness-of-fit metrics (mean square error, MSE and coefficient of determination, *R* ^2^) are shown in Figure 4. After the split, the σ value of each Gaussian curve becomes smaller than the previous distribution (Figure S3); meanwhile, the MSE of the separation curves is lower than 0.0002 with the *R* ^2^ higher than 0.85 (Figure 4B), which verifies the dependability of the fitting. Depending on the mean *N*_molecules_ for H_2_O_2_ obtained from splitting Gaussian curves in Figure 4A,C, the concentration inside SGs is calculated to be 2.37 M (0–50 nm SGs; 50 nm nanopipettes), 71.00 mM (50–200 nm), 8.66 mM (200–500 nm), 2.98 mM (500–750 nm), and 5.45 mM (750–1000 nm). The concentration in the smallest SGs is extraordinarily high, but in extremely small structures, concentrations can sometimes be at the molar level owing to the small number of molecules required. This has physiological precedence in vesicles, where, owing to the protein-dense core, certain small vesicles can have concentrations near 2 M in vesicles thought to have a radius of about 45 nm.^25^ The relationship of the H_2_O_2_ concentration with the different volumes and areas of the SGs from Figure 4C are shown in Figure S5. The H_2_O_2_ gradient progressively decreases from 2.37 M in <50 nm SGs to millimolar levels for the larger SGs. This trend is different from that of vesicles, where the vesicular redox species concentration is usually independent of vesicle size.^26,27^ The phenomenon implies a critical role of granule size in SG compartment redox activity, which highly resembles the connection between the concentration of H_2_O_2_/surface electric field of microdroplets and their size,^28^ indicating that the size or the surface electric field of the SG plays a role in the generation process of H_2_O_2_.

**Figure 4:**
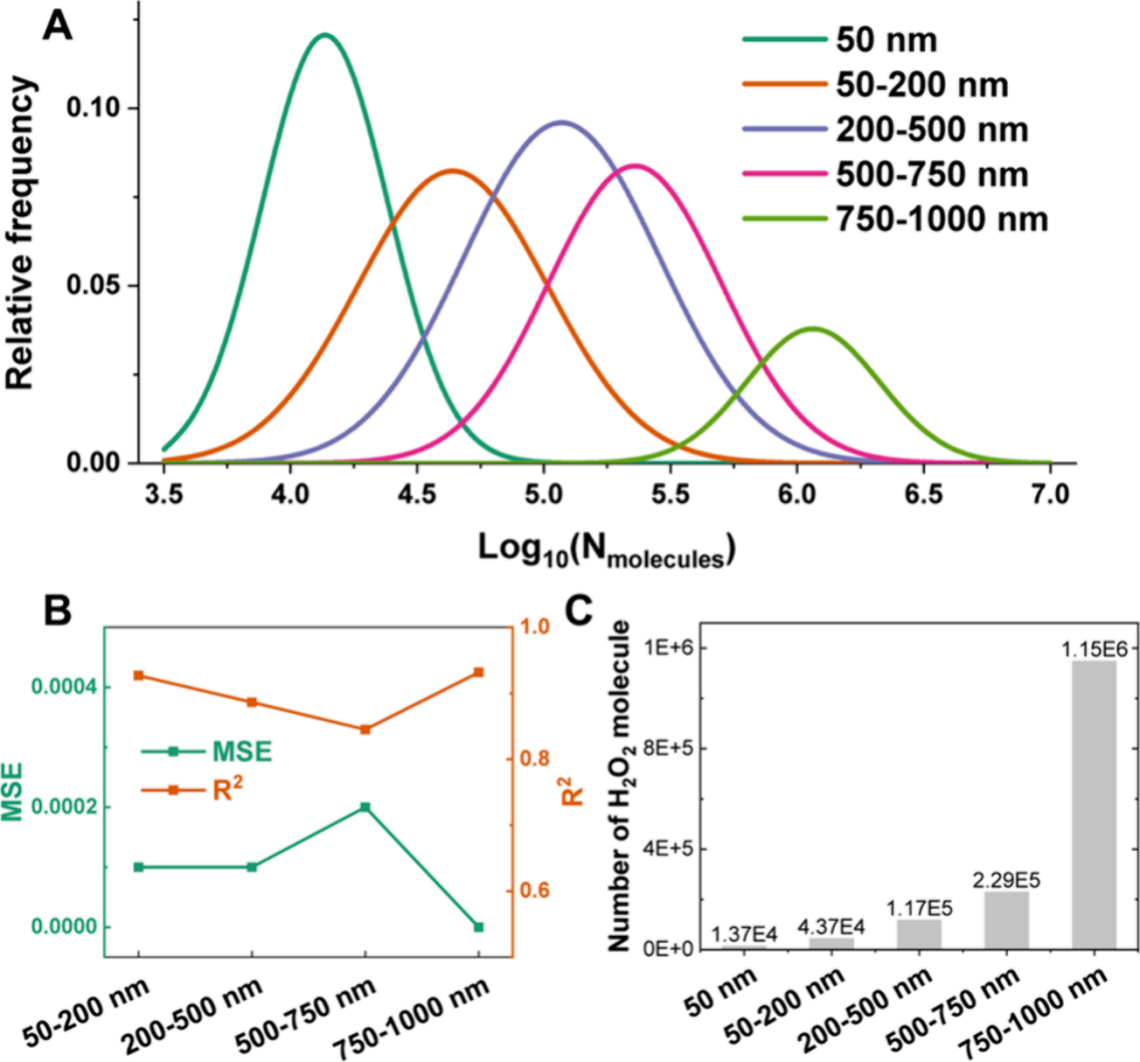
Distribution of molecule number within the specific size ranges of SGs. (A) Isolated Gaussian curves from the total frequency histogram in Figure 2A and (B) their goodness-of-fit parameters (MSE and *R* ^2^) (B). (C) Relationship between the median of Gaussian curves and the size ranges of SGs.

In addition to the use of SGIEC in vitro, two sizes (50 and 750 nm) of Pt-CNTNPs were employed to conduct ISGIEC of SGs in living cells. Figure 5A shows a representative ISGIEC amperometric trace using a 50 nm diameter Pt-CNTNP at 700 mV. The number of molecules and the half-peak width (*t*_1/2_) from each ISGIEC test were statistically calculated and averaged as shown in Figure 5B,C. Both *N*_molecules_ and *t*_1/2_ for H_2_O_2_ show the same patterns as those in the extracellular SGIEC experiment. There are no significant differences in *t*_1/2_ between intracellular and isolated SGs for both 50 and 750 nm-diameter electrodes (Figure S6). However, discrepancies were identified when comparing the *N*_molecules_ for the same-sized electrodes in the SGIEC and ISGIEC experiments (Figure 5D). The molecular number for the 50 nm electrode is marginally larger for the SGIEC and is not significantly different at *p* = 0.05. This might result from the difference in buffer solution in vitro and in the cell. However, the number for the 750 nm electrode in the ISGIEC data is considerably smaller than that for SGIEC. We considered that macromolecular crowding inside cells, with macromolecules like RNA and proteins occupying roughly 20–30% of the cytoplasmic volume, might generate a spatially unreachable space for numerous cytoplasmic components^29^ and might be capable of modifying the molecular diffusion and collision frequencies and also affecting the binding affinity of interacting biological assemblies.^30,31^ Additionally, some reports suggest a decreased activity of the water solvent because of the alterations in the hydrogen-bonding structuring of water in crowded environments.^32^ To validate this hypothesis, 5% PEG8000 was added to the SGIEC experiment to simulate the intracellular macromolecular crowding condition with isolated SGs. PEG8000 is a commonly used crowding agent to mimic the cellular environment in vitro; usually adding 10% PEG8000 will make the viscosity in the solution system reach the physiological range;^33^ however, with a temperature decrease to 277 K, the viscosity of PEG8000 increases about 2-fold.^34^ Thus, 5% PEG was selected to simulate the physiological environment of the cell interior under the experimental conditions. Typical amperometric traces for the experimental and control groups for two-sized electrodes were obtained. The distribution pattern between the number of molecules in SGs and their sizes is continuous, so we chose nanopore electrode sizes at either end of the size range to test the SGs: 50 and 750 nm, allowing detection of most of the SGs over their range. SGs of intermediate sizes can also enter the 750 nm electrode for detection, so their corresponding phenomena have been included in the analysis data of the 750 nm electrode. The amperometric traces are shown in Figure S7, and the average *N*_molecules_ are presented in Figure 5D. After adding PEG8000, the number of molecules in <50 nm SGs decreased slightly with no significant variance in comparison with ISGIEC and control, but the average *N*_molecules_ in <750 nm SGs decreased significantly to the ISGIEC level. These data, added to the disruption of the signal with catalase^10^ and electrochemical data,^13^ support the hypothesis that the ROS formed at SGs is H_2_O_2_ and lean toward macromolecular crowding exerting a role in determining the concentration of H_2_O_2_ formation at SGs. These data also strongly support the hypothesis that the formation of H_2_O_2_ is at the interface of the SG with the solution rather than internally.^13^ Through the coordinated regulation of multiple physical aspects of the nanoenvironment, altering the interfacial electric field of SGs by controlling the molecular diffusion, affecting the binding force of biomolecular interactions, and decreasing the water solvent activity, the H_2_O_2_ content in large-sized SGs is eventually lowered by macromolecular crowding.

**Figure 5:**
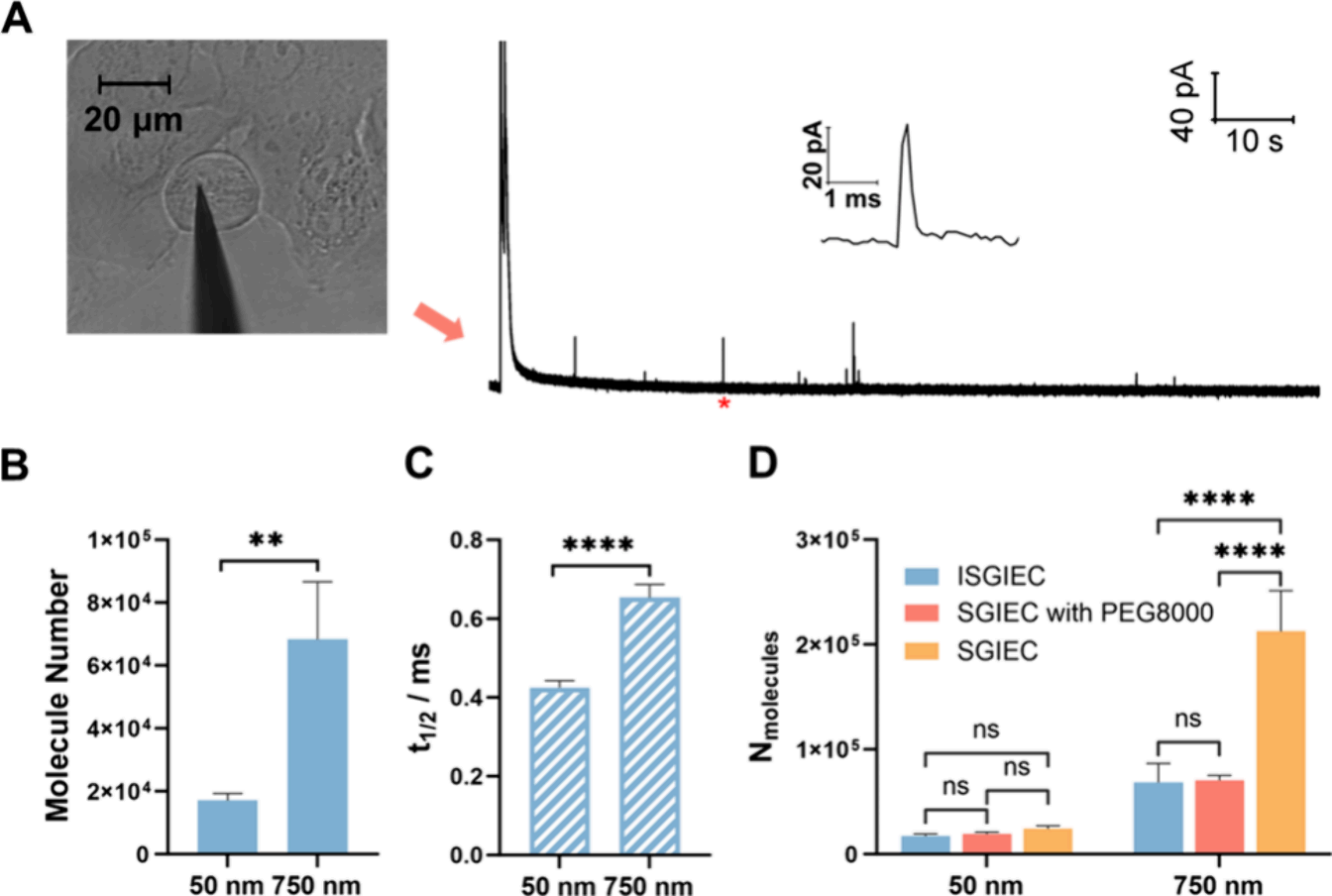
(A) Micrograph of a 50 nm diameter Pt-CNTNP in a U2OS cell and representative ISGIEC measurement trace at 700 mV vs Ag/AgCl. Characteristic current transient shown with red asterisk. Electrode inserted at the red arrow. Average number of molecules (B) and half-peak widths *t*_1/2_ (C) in a single SG tested by two sizes of Pt-CNTNPs (50 and 750 nm). (D) Average number of molecules using ISGIEC and SGIEC (with and without PEG8000) methods at 50 and 750 nm electrodes. Error is SEM. Two-tailed Mann–Whitney rank-sum test on Figure 5B,C. One-way ANOVA on Figure 5D ranks: ns *p* > 0.5, **p < 0.01, and ****p < 0.0001. ISGIEC data were collected from three cultures of U2OS cells and 25 Pt-CNTNPs. SGIEC data were collected from three isolations of SGs from U2OS cells and 54 Pt-CNTNPs in the experimental and control groups.

In conclusion, we have performed SGIEC and ISGIEC measurements with novel Pt-CNTNPs of different sizes. The size exclusion in different nanopipettes allows us to determine the relationship between H_2_O_2_ content/production and measurement kinetics at different SG sizes. A Python fitting program was used to sort the Gaussian distributions of the H_2_O_2_ *N*_molecules_ of different size ranges, allowing the concentration of H_2_O_2_ at SGs within a specific size range to be obtained. We used PEG8000 in vitro and simulations to suggest a macromolecular crowding mechanism for the difference in *N*_molecules_ detected within and outside the cells. These conclusions suggest that the generation mechanism of H_2_O_2_ at SGs is mainly at the SG-solution interface, perhaps related to an interfacial electric field across this interface, as suggested previously.^35,36^

We have established an oxidative stress regulation model for SGs that is dependent on the sizes of these structures. This suggests a new treatment concept for cellular stress by interfering with the volume of the SGs. Here, the cellular stress response can be modulated to treat related diseases such as neurodegenerative diseases (such as Alzheimer’s disease) and cancer. Simultaneously, it offers a novel perspective for comprehending the chemical regulatory mechanism of LLPS and might influence the domains of precision medicine, nanocatalysis, and basic cell biology.

## Supplementary Material



## Notes

Additional experimental details; CVs of H_2_O_2_ at Pt–Au NPs-CNTNP, Pt-CNTNP, CNTNP, and CNP; TEM characterization of Pt–Au NPs-CNTNPs with different sizes; variation of standard deviation values for each distribution by nanopore size before and after analytical treatment; relationship of the average half-peak width with the size of the electrode; relationship of the hydrogen peroxide concentration with the different sizes of the SGs; average *t*_1/2_ from ISGIEC and SGIEC measurement at 50 and 750 nm electrodes; representative amperometry traces obtained in different electrodes of different conditions; size distribution of separated SGs tested by dynamic light scattering; CVs with different concentration of H_2_O_2_ at small electrodes; CVs at different pH value and different concentration of H_2_O_2_ using Pt-CNTNPs (PDF)

## ABBREVIATIONS

SGs: stress granules
LLPS: liquid–liquid phase separation
Pt −CNTNPs: platinized carbon nanotube nanopipettes
SGIEC: SG impact electrochemical cytometry
ISGIEC: intracellular SGIEC
H2O2: hydrogen peroxide
ROS: reactive oxygen species
VIEC: vesicle impact electrochemical cytometry
CNPs: carbon nanopipettes
CNT-NPs: carbon nanotube nanopipettes
TEM: transmission electron microscope
Pt-CNPs: platinized CNPs.
