# Supplementary material for: Correlating Molecule Counts with Stress Granule Size Using Novel Platinized Carbon Nanopore Electrodes
Source: Anal Chem. 2026 Jun 2;98(23):17153–60. doi: 10.1021/acs.analchem.6c01191 (PMC13276837; doi:10.1021/acs.analchem.6c01191)
Supplement: Supplementary file 1 [file ac6c01191_si_001.pdf]

Supporting information for

Correlating Molecule Counts with Stress Granule Size Using Novel Platinized Carbon Nanopore Electrodes

Yue Wang[a, b], Chaoyi Gu[a], Hui Gu[a, c], Yingjie Zhao[b], Dengchao Wang[b], Andrew G. Ewing*[a]*

[a] Department of Chemistry and Molecular Biology, University of Gothenburg, 41390 Gothenburg (Sweden)

[b] School of Chemical Sciences, University of Chinese Academy of Sciences, 10049 Beijing (China)

[c] Department of Chemistry and Chemical Engineering, Hunan University of Science and Technology, 411201 Xiangtan (China).

*Email: andrew.ewing@chem.gu.se, wangdengchao@ucas.ac.cn

1. EXPERIMENTAL

1.1. Python program for nonlinear fitting

2. FIGURES

Figure S1. Cyclic voltammograms with 1 mM H_2O_2 in 10 mM PBS buffer at Pt-Au NPs-CNTNP, Pt-CNTNP, CNTNP and CNP. (scan rate=0.1 V/s)

Figure S2. Typical TEM characterization images of Pt-Au NPs-CNTNPs with different sizes (A) and frequency histograms of the electrode sizes prepared with the same parameter (B). n=25, error is SEM.

Figure S3. Variation of standard deviation (σ) values for each distribution by nanopore size before and after analytical treatment of the data to remove smaller events from larger nanopore experiments (splitting the Gaussian curves from data in Figure 3A). This results in a change in the σ when comparing the uncorrected data (Figure 3A, green line) and the corrected data (Figure 4A, red line) over the range of different sizes of electrodes.

Figure S4. Relationship of the average half-peak width with the size of the electrode. The confidence band of $t_{1/2}$ is at level 0.95.

Figure S5. Relationship of the hydrogen peroxide concentration with the different volumes (A) and areas (B) of the SGs from Figure 4C. The insets were obtained after deleting the data of SGs with a diameter of 50 nm.

Figure S6. The average $t_{1/2}$ from ISGIEC and SGIEC measurement at 50 nm and 750 nm electrodes. n=47, error is SEM. One-way ANOVA on ranks: ns $p > 0.5$.

Figure S7. Representative amperometry traces obtained in 50 nm Pt-Au NPs-CNTNPs and 750 nm Pt-Au NPs-CNTNPs with/without the addition of 5% PEG8000 at +700 mV vs. Ag/AgCl. Characteristic current transients with red asterisks are magnified in insets. Each spike represents a single SG collision event.

1 EXPERIMENTAL

1.1. Python program for nonlinear fitting

This script performs a hybrid model fitting combining a pre-defined reference curve and a Gaussian function to characterize experimental nanoparticle size distributions. The function $f_{known}(x)$ is derived from the composite experimental data of electrodes at a smaller scale.

```
# Module Import
import numpy as np
from scipy.interpolate import interp1d
from scipy.optimize import curve_fit
import matplotlib.pyplot as plt

# Data Loading & Preprocessing
known_data = np.loadtxt('50 nm.txt')
x_known = known_data[:, 0]
y_known = known_data[:, 1]
known_function = interp1d(x_known, y_known, kind='linear', fill_value="extrapolate")

# Hybrid Model Definition
from scipy.optimize import curve_fit
def combined_model(x, scale, A, mu, sigma):
    known_part = scale * known_function(x)
    gaussian = A * np.exp(-(x - mu)**2 / (2 * sigma**2))
    return known_part + gaussian

# Experimental Data Preparation
x_data = np.linspace(3.5, 7.0, 36)
y_data = np.array([0.003921569, 0, 0, 0.011764706, 0.039215686, 0.050980392, 0.070588235, 0.070588235, 0.090196078, 0.101960784, 0.066666667, 0.08627451, 0.098039216, 0.101960784, 0.031372549, 0.047058824, 0.043137255, 0.035294118, 0.019607843, 0.011764706, 0.007843137, 0.003921569, 0, 0, 0.003921569, 0, 0, 0.003921569, 0, 0, 0, 0, 0, 0, 0, 0])

# Model Fitting
initial_guess = [0.4, 0.6, 4.7, 0.35]
params, covariance = curve_fit(
    combined_model,
    x_data,
    y_data,
    p0=initial_guess,
    bounds=([0, 0, 3.5, 0.1], [2, 5, 7.0, 2.0])
)
scale_fit, A_fit, mu_fit, sigma_fit = params
y_combined_fit = combined_model(x_data, *params)
gaussian_fit = A_fit * np.exp(-(x_data- mu_fit)**2 / (2 * sigma_fit**2))
```

```
# Result Saving
np.savetxt('200 nm.txt',
        np.column_stack((x_data, y_combined_fit)),
        fmt='%.6f',
        delimiter=' ')
np.savetxt('50-200 nm.txt',
        np.column_stack((x_data, gaussian_fit)),
        fmt='%.6f',
        delimiter=' '
)

# Goodness-of-Fit Evaluation
y_fit = combined_model(x_data, *params)
residuals = y_data - y_fit
mse = np.mean(residuals**2)
ss_total = np.sum((y_data - np.mean(y_data))**2)
ss_residual = np.sum(residuals**2)
R_squared = 1 - (ss_residual / ss_total)

# Results
print(f"alpha: {scale_fit:.2f}, amplitude: {A_fit:.2f}, mean: {mu_fit:.2f}, standard deviation: {sigma_fit:.2f}")
print(f"mean square error (MSE): {mse:.4f}")
print(f"coefficient of determination (R²): {R_squared:.4f}")
import matplotlib.pyplot as plt
plt.scatter(x_data, y_data, label='experiment data', alpha=0.6)
plt.plot(x_data, combined_model(x_data, *params), 'r-', label='final fit curve')
plt.plot(x_known, y_known, 'g--', label='origin curve A')
plt.plot(x_known, scale_fit * y_known, 'b--', label='changed curve A')
plt.plot(x_data, gaussian_fit, 'b--', label='fit curve B')
plt.legend()
plt.show()
```

2 FIGURES

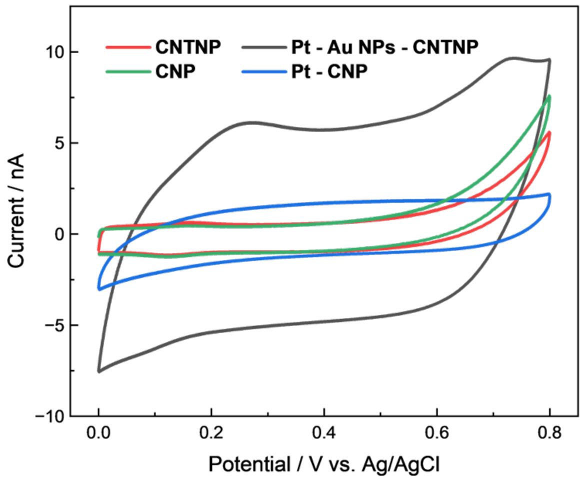

Figure S1. Cyclic voltammograms with 1 mM H_2O_2 in 10 mM PBS buffer at Pt-Au NPs-CNTNP, Pt-CNTNP, CNTNP and CNP. (scan rate=0.1 V/s).

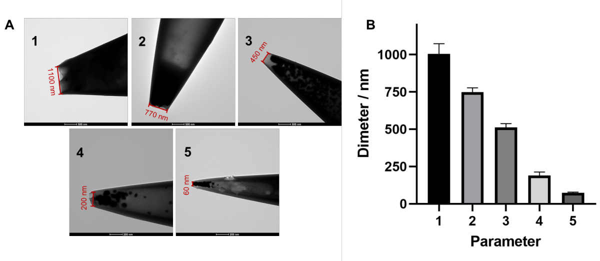

Figure S2. Typical TEM characterization images of Pt-Au NPs-CNTNPs with different sizes (A) and frequency histograms of the electrode sizes prepared with the same parameter (B). n=25, error is SEM.

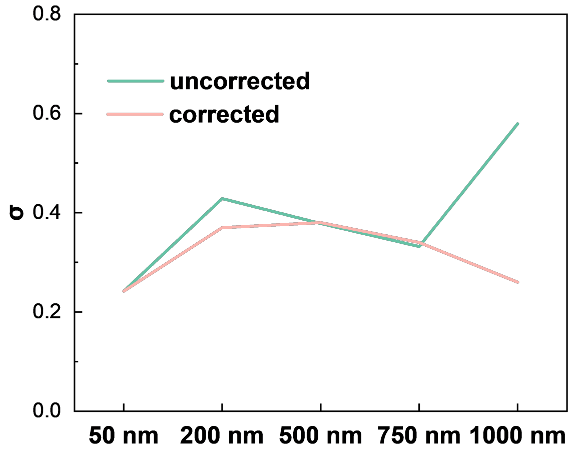

Figure S3. Variation of standard deviation (σ) values for each distribution by nanopore size before and after analytical treatment of the data to remove smaller events from larger nanopore experiments (splitting the Gaussian curves from data in Figure 3A). This results in a change in the σ when comparing the uncorrected data (Figure 3A, green line) and the corrected data (Figure 4A, red line) over the range of different sizes of electrodes.

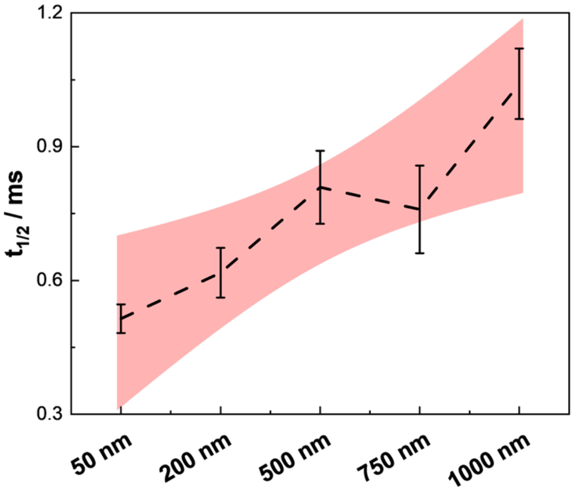

Figure S4. The relationship of the average half-peak width with the size of the electrode. The confidence band of $t_{1/2}$ is at level 0.95.

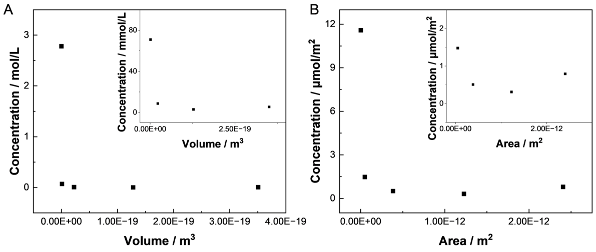

Figure S5. Relationship of the hydrogen peroxide concentration with the different volumes (A) and areas (B) of the SGs from Figure 4C. The insets were obtained after deleting the data of SGs with a diameter of 50 nm.

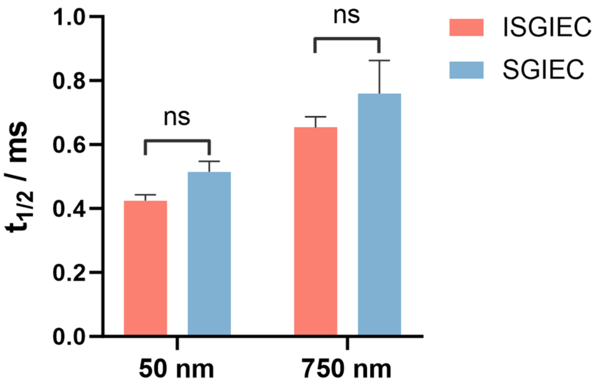

Figure S6. The average $t_{1/2}$ from ISGIEC and SGIEC measurement at 50 nm and 750 nm electrodes. n=47, error is SEM. One-way ANOVA on ranks: ns $p > 0.5$.

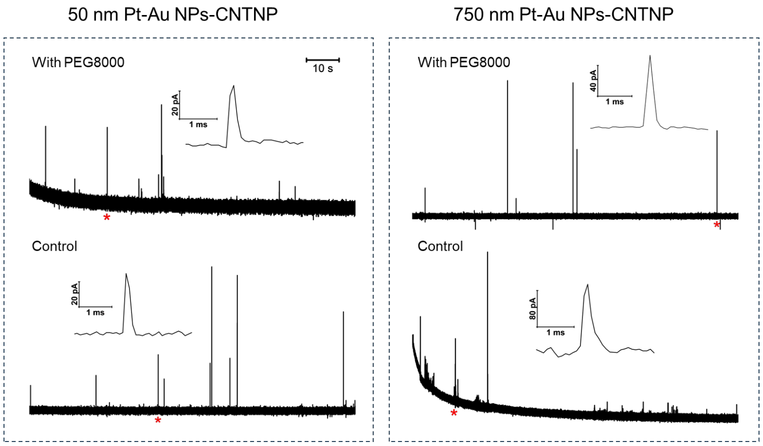

Figure S7. Representative amperometry traces obtained in 50 nm Pt-Au NPs-CNTNPs and 750 nm Pt-Au NPs-CNTNPs with/without the addition of 5% PEG8000 at +700 mV vs. Ag/AgCl. Characteristic current transients with red asterisks are magnified in insets. Each spike represents a single SG collision event.
